# CD79B and MYD88 Mutations in Splenic Marginal Zone Lymphoma

**DOI:** 10.1155/2013/252318

**Published:** 2013-01-10

**Authors:** Gunhild Trøen, Abdirashid Warsame, Jan Delabie

**Affiliations:** ^1^Department of Pathology, Oslo University Hospital, The Norwegian Radium Hospital, 0310 Oslo, Norway; ^2^Faculty of Medicine, University of Oslo, 0316 Oslo, Norway

## Abstract

The mutation status of genes involved in the NF-**κ**B signaling pathway in splenic marginal zone lymphoma was examined. DNA sequence analysis of four genes was performed: CD79A, CD79B, CARD11, and MYD88 that are activated through BCR signaling or Toll-like and interleukin signaling. A single point mutation was detected in the CD79B gene (Y196H) in one of ten SMZL cases. Additionally, one point mutation was identified in the MYD88 gene (L265P) in another SMZL case. No mutations were revealed in CD79A or CARD11 genes in these SMZL cases. Neither were mutations detected in these four genes studied in 13 control MZL samples. Interestingly, the two cases with mutations of CD79B and MYD88 showed increased numbers of immunoblasts spread among the smaller and typical marginal zone lymphoma cells. Although SMZL shows few mutations of NF-**κ**B signaling genes, our results indicate that the presence of these mutations is associated with a higher histological grade.

## 1. Introduction

Marginal zone lymphoma (MZL) is a non-Hodgkin lymphoma that likely develops from B-lymphocytes in the marginal zone of secondary lymphoid tissue. There are three subtypes of marginal zone B-cell lymphoma (MZL) [1]: nodal, extranodal, and splenic marginal zone lymphoma, arising in the lymph node, mucosa, and the spleen, respectively. Splenic marginal zone lymphoma (SMZL) is an indolent, low grade B-cell lymphoma primarily characterized by splenomegaly with variable involvement of lymph nodes, bone marrow, peripheral blood, and other organs. It accounts for less than 1% of non-Hodgkin lymphoma [2]. The normal splenic marginal zone contains both memory B cells and naive B cells. In parallel, unmutated as well as mutated immunoglobulin heavy chain genes are found [3–5].

Gene expression profiling has revealed aberrant NF-*κ*B signaling in several lymphoma types such as diffuse large B-cell lymphoma (DLBCL) [6, 7], Hodgkin lymphoma [8], and SMZL [9]. NF-*κ*B is a transcription factor that regulates different cellular processes, such as cell growth and survival [10] and is activated when normal B-cells respond to antigen. In one subtype of DLBCL, of activated B-cell origin (ABC), NF-*κ*B signaling is constitutively activated due to mutations of important B-cell receptor (BCR) signaling genes [11]. These include mutations of the gene for caspase recruitment domain-containing protein 11 (CARD11) in 10% [12] and mutations and/or deletions of CD79, an essential signaling subunit of the BCR in 21% of ABC DLBCL [13]. Of interest, Ngo et al. have described oncogenic MYD88 mutations [14]. MyD88 is an adaptor protein involved in Toll-like receptor signaling leading to NF-*κ*B activation in both ABC DLBCL (29%) and in MALT lymphoma (9%). MYD88 mutations in SMZL were hitherto not studied. Interestingly, Rossi et al. demonstrated mutations and copy number alterations of genes such as TNFAIP3, IKBKB, BIRC3, TRAF3, and MAP3K14, involved in both the canonical and noncanonical NF-*κ*B pathways in about 20% of SMZL [15]. These authors found no mutations in CARD11 and MYD88. However, in a more recent study both CARD11 mutations and MYD88 mutations have been detected in, respectively, 8.8% and 13% of SMZL [16]. The reason for the discrepancies between the two studies is unclear. The aim of this study was to analyze somatic mutations in additional genes, specifically CD79A and B, and study CARD11 and MYD88 gene mutations in our cases of SMZL.

## 2. Methods

Tissue samples of 10 cases of SMZL and 13 control cases of other MZL types, 7 nodal and 6 extranodal MALT-type, were selected from the archives of the Department Pathology at The Norwegian Radium Hospital, Oslo University Hospital, Norway. All studied cases were reviewed to confirm diagnoses. The study was approved by the Regional Committee for Research Ethics.

DNA was isolated from frozen tissue using the EZ1 tissue kit (Qiagen, Hilden, Germany). PCR was carried out using AmpliTaq Gold polymerase (Applied Biosystems, Weiterstadt, Germany) according to the supplier's instructions and using the following conditions: 94°C for 5 min followed by 34 cycles of denaturation 30 s at 94°C, annealing 30 s at 60°C (or 58°C), and extension 45 s at 72°C. PCR primers were used as described in Table 1. The primer pairs for CD79A (exon 4 and 5, NM_001783), CD79B (exon 5 and 6, NM_000626), and MYD88 (part of exon 5, NM_002468) were designed using Primer-BLAST. The primer pairs used for CARD11 exon 5–10 (NM_032415) were as described in Lenz et al. (exon 5–10 is equal to exon 3–8 in their publication) [12]. The primer pairs used are shown in Table 1. The PCR products were verified on an Agilent 2100 Bioanalyzer (Agilent Technologies) and an aliquot of the products was directly sequenced from both ends on a 3130 Genetic Analyzer (Applied Biosystems) using BigDye Terminator v1.1 (Applied Biosystems). A single nucleotide polymorphism in one amino acid position (265) of the MYD88 gene was detected using PCR and SNaPshot multiplex kit (Applied Biosystems) and analyzed with both forward and reverse primers by 3130 Genetic Analyzer and GeneMapper 4.1 Software (Applied Biosystems). 

## 3. Results

**Figure 1:**
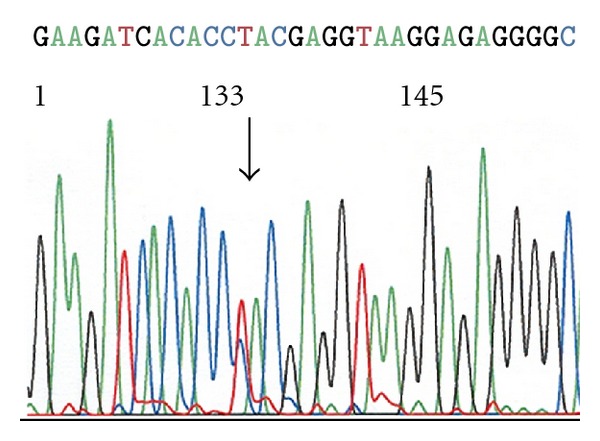
Electropherogram illustrating mutation Y196H (c.586T>C) of the CD79B gene in one case of SMZL.

**Figure 2:**
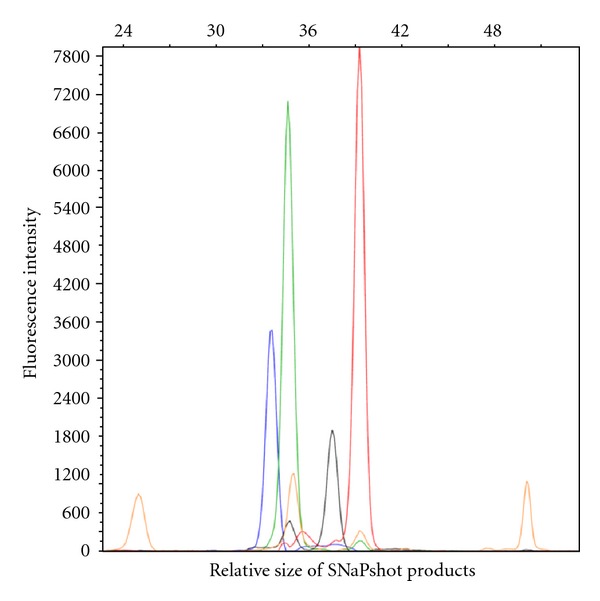
Electropherogram of SNaPshot products illustrating the heterozygous loci detected in the MYD88 gene (c.794T>C, L265P) in one case of SMZL. The plot shows the relative fluorescence intensity versus the measured size in nucleotides of the products relative to the GeneScan-120 LIZ internal size standard (orange peaks). Bases are represented by the following colours: T = red, wild type; C = black, mutated; A = green, wild type G = blue, mutated.

A point mutation in the CD79B gene (c.586T>C, Y196H) was detected in one SMZL case (Figure 1) whereas a MYD88 mutation (c.794T>C, L265P) was detected in another case (Figure 2). The CD79B mutation affected the first tyrosine of the CD79B immunoreceptor tyrosin-based activation motif (ITAM). No mutations were detected in the CD79A gene or in the CARD11 gene in any of the SMZL cases (Table 2). No mutations of CD79A, CD79B, CARD11, and MYD88 were detected in the 13 control MZL samples. 

**Figure 3:**
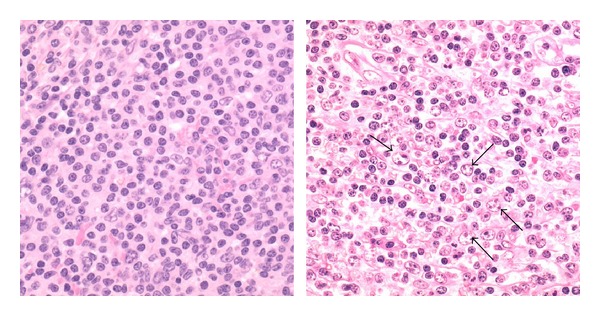
The left panel illustrates the lymphoma histology of a SMZL case without mutations in CD79A, CD79B, CARD11, or MYD88. The spleen shows homogenous infiltration with small cells with abundant clear cytoplasm (H&E, 400x). The right panel illustrates the lymphoma histology case with a CD79B mutation. The spleen shows infiltration with small lymphoid cells as well as with larger immunoblast-like cells (arrows, H&E, 400x).

Of interest, the two SMZL cases with mutations of CD79B and MYD88, respectively, showed increased numbers of immunoblasts spread among the smaller and typical marginal zone lymphoma cells (Figure 3).

## 4. Discussion

A low frequency of mutations in four NF-*κ*B-signaling genes in SMZL was detected. Out of 10 cases of SMZL, only one mutation was found in CD79B and one mutation in MYD88 and none in CD79A and CARD11. This is in contrast to ABC DLBCL showing CD79B mutations in 21%, MYD88 mutation (L265P) in 29%, and CARD11 mutations in 10% of the cases [12–14]. However, no CD79B mutations were detected in 16 gastric MALT lymphoma cases analyzed in the same study [13]. Ngo et al. found a similar low frequency of MYD88 mutation (9%) in gastric MALT lymphomas [14] as we found in SMZL.

The mutation detected in CD79B replaces the first tyrosine in the ITAM, a critical residue, by another amino acid. Interestingly, this particular mutation is also found in ABC DLBCL. This mutation increases the surface BCR expression, inhibits Lyn kinase activity, and enhances activation of NF-*κ*B in ABC DLBCL [13]. The MYD88 L265P mutation detected in one of the SMZL cases occurs at a residue in the TIR domain that is important for protein complex assembling and supports cell survival by activating NF-*κ*B and JAK-STAT signaling pathways [14]. Although the somatic origin of the two mutations detected in our study could not be verified, these mutations have been shown by others to be functionally significant. It is therefore likely that mutations leading to amino acid substitutions demonstrated in two of the SMZL samples represent somatic mutations. 

A recent report has described mutations of multiple genes that cause deregulation of NF-*κ*B in DLBCL [11]. Of these the NF-*κ*B negative regulator TNFAIP3 (A20) shows inactivation mutations and/or deletions in about 30% of patients, thus contributing to NF-*κ*B activation. More importantly, A20 is also inactivated in 19% if MZL, however with a frequency of only 8% in SMZL [17]. A low mutation frequency in two of the four NF-*κ*B-related genes studied is also reported by a recent study of a large series of SMZL [15]. Out of 101 cases of SMZL no mutations or copy number abnormalities of CARD11 and MYD88 were revealed, while CD79A and B were not studied. However, out of the other 20 genes studied, 5 of these genes showed mutations in 20% of SMZL cases. Yan and colleagues [16] found a similar level of MYD88 mutation (13%) as in our study, however no mutations were detected in CD79A or CD79B in their series of 57 SMZL cases. 

The cases with CD79B mutation and MYD88 mutation, respectively, showed increased numbers of immunoblast-like cells compared with the cases without mutation. This observation is of interest and might indicate that both mutations are associated with higher histological grade and therefore with risk of transformation. However, larger series with clinical follow-up data are needed to confirm the latter.

In this study we found mutations in NF-*κ*B-signaling genes in SMZL, including for the first time in CD79B. However, mutations occur in only few cases. It is therefore likely that yet other genetic aberrations of signaling genes may contribute to the constitutive activation of NF-*κ*B in SMZL. 

## Figures and Tables

**Table 1:** Primer pairs used for amplification and sequencing of CD79A, CD79B, CARD11, and MYD88 genes in SMZL.

	Forward	Reverse
CD79A exon 4	cat cca gga ggg tct gaa ag	ccc taa cac aac tgc ccc ta
CD79A exon 5	agg tgt cag ggt gct gat gt	ccc act ggg gga ata tga ct
CD79B exon 5	tct tgc aga atg cac ctc ac	gca gcg tca cta tgt cct ca
CD79B exon 6	tac gag gta agg aga ggg gc	aga caa atg gca gct ctg gt
CARD11 exon 5	gtc acc ctg gcg gag tag cc	cag tgc ctc gtg ggc aga gt
CARD11 exon 6	ctg gag aag gtt tct tgg agc	aca ccc tgg cag gtt cat c
CARD11 exon 7	ccc agg ccc tca tct ggt tg	ccc agg ata cgc cca agc aa
CARD11 exon 8	tcc cct atg tta cct ggt ctg tag tg	gcc tgt gac ttc caa aaa agc c
CARD11 exon 9	cct cag tgc cct cat ctg taa aat g	caa agg aca agg agc cat tca ttg
CARD11 exon 10	agc gag tcg cag gat ttc ca	cca gaa gcc tgg gag gag ga
MYD88 PCR	tgc agg tgc cca tca gaa gcg	cag aca gtg atg aac ctc agg atg c
MYD88	ccc ccc ccc c	
SNaPshot	agg tgc cca tca gaa gcg ac	cct tgt act tga tgg gga tc

**Table 2:** Detected mutations in CD79A, CD79B, CARD11, and MYD88.

Gene analyzed	SMZL cases	Other MZL cases
	Detected mutations/ cases analyzed	Detected mutations/ cases analyzed
CD79A (exon 4 and 5)	0/10	0/13
CD79B (exon 5 and 6)	1/10	0/13
CARD11 (exon 5–10)	0/10	0/13
MYD88 (one hotspot)	1/10	0/13
